# Results of the Max Page muscle sliding operation for the treatment of Volkmann’s ischemic contracture of the forearm

**DOI:** 10.1007/s10195-012-0212-0

**Published:** 2012-08-02

**Authors:** Pulak Sharma, M. K. S. Swamy

**Affiliations:** 1 Central Institute of Orthopedics, VMMC and Safdarjung Hospital, New Delhi, India; 2 A2-403, Glaxo Appt., Mayur Vihar Phase 1, New Delhi, 110091 India

**Keywords:** Max Page release, Volkmann’s contracture, Tight external bandage

## Abstract

**Background:**

Volkmann’s ischemic contracture is a less common but crippling condition affecting the extremities. Once the condition sets in, the prognosis always remains guarded, even after long and intensive physiotherapy and various restorative surgical techniques. This study was undertaken to evaluate the long-term functional results of the Max Page muscle slide operation in patients with Volkmann’s ischemic contracture of the forearm of moderate degree (Tsuge classification).

**Materials and methods:**

Nineteen patients treated between 1997 and 2009 were evaluated. The functional outcome (measured as the dexterity score, hand grip strength, sensibility, and appearance) was analyzed postoperatively. The pre- and postoperative values were compared using a paired *t* test. The final results were graded as good, fair, and poor.

**Results:**

The average age at the time of presentation was 18 years (range 3–25 years). Tight external splintage for injuries around elbow and forearm was the primary factor. The mean period of follow-up was 3.53 years. Fifteen patients were able to achieve good functional results. Three had fair and one had poor results. All three variables showed significant improvements postoperatively. Wound dehiscence was the most common complication. One patient needed a second surgery to restore good hand function.

**Conclusion:**

The Max Page muscle sliding operation to treat Volkmann’s ischemic contracture of moderate degree gives good functional results. The procedure is simple and easy to perform. Adequate muscle release and proper postoperative physiotherapy are key to achieving good results.

## Introduction

In 1881, Volkmann [1] described a condition involving muscle ischemia and necrosis that subsequently led to fibrosis and contracture of the forearm. He had attributed the contracture to an interruption of the arterial blood supply caused by the application of tight compression bandages to the injured limb. In 1909, Thomas [2] collected 107 cases that had been reported in the literature, and among these were cases in which no splint or bandage had been applied. Brooks [3] described a similar condition and believed that venous obstruction was the main factor involved in contracture formation. Arterial spasm or injury were indicated as causes by Leriche [4] and Griffiths [5].

It is now well recognized that ischemic contractures can develop from many different injuries, as long as the injuries cause swelling of the soft tissues that are contained in relatively nondistensible osseofascial compartments [6]. As a result of this swelling, intramuscular pressure is elevated to a magnitude sufficient to occlude capillary perfusion. The compartments with the least ability to expand are most severely affected by this ischemic insult. Because the deep flexor compartment of the forearm lies next to the bone, it is the first and most severely affected [7]. Variable levels of necrosis and fibrosis, though to a lesser degree, may also be evident in the flexor digitorum superficialis and the superficially located muscles such as the wrist flexors. The muscle degeneration which follows is most marked in the middle, and its extent decreases peripherally, so that a so-called ellipsoid-shaped infarct (as described by Seddon [7]) may be the end result. Nerve trunks running in the ischemic zone also suffer damage; this is initially caused by ischemia but is later aggravated by the subsequent muscle fibrosis.

Despite our improved understanding of the pathogenesis, diagnosis, and treatment of compartment syndrome of the forearm, this condition can easily be missed. Precise knowledge of the symptoms is required to recognize the syndrome in time. Immediate identification of the compartment syndrome and its early treatment is mandatory to avoid its devastating consequences, such as Volkmann’s ischemic contracture. Once the contracture sets in, the prognosis is always guarded, even after long and intensive physiotherapy and various restorative surgical techniques.

Various treatment options have been mentioned in the literature, ranging from splinting and passive stretching to osteotomy, excision of fibrotic tissue, muscle slide, neurolysis, tendon transfer, and free functional muscle grafts. Once a diagnosis has been made, ischemic contractures are classified according to the severity of involvement, and the suitable treatment option is selected.

The aim of this study was to review the functional results of the Max Page muscle slide operation in patients with Volkmann’s ischemic contracture of moderate degree, as per the Tsuge classification [8] (Table 1).

**Table 1:** Tsuge classification of Volkmann’s ischemic contracture

Type	Affected muscles	Neurological	Finger position
Mild	Flexor digitorum profundus	No or minimal loss of sensibility	Contracture of two or three fingers
Moderate	Flexor digitorum profundus, flexor pollocis longus, and parts of superficial flexor muscles	Loss of sensibility in (parts of) the hands	All fingers, the thumb, and often the wrist are affected
Severe	All flexor muscles and parts of the extensor muscles	Serious loss of sensibility or motor function	Claw hand

## Materials and methods

During the 12-year period between 1997 and 2009, 29 patients with Volkmann’s ischemic contracture were treated. Six patients who had mild deformity were treated by tendon lengthening of the flexor digitorum profundus, whereas four patients with severe deformity were treated by carpectomy. Nineteen patients who had moderate-degree Volkmann’s ischemic contracture (Tsuge classification) were treated by muscle slide and are reviewed in this paper. The study was authorized by the local ethical committee and was performed in accordance with the ethical standards of the 1964 Declaration of Helsinki as revised in 2000.

**Fig. 1:**
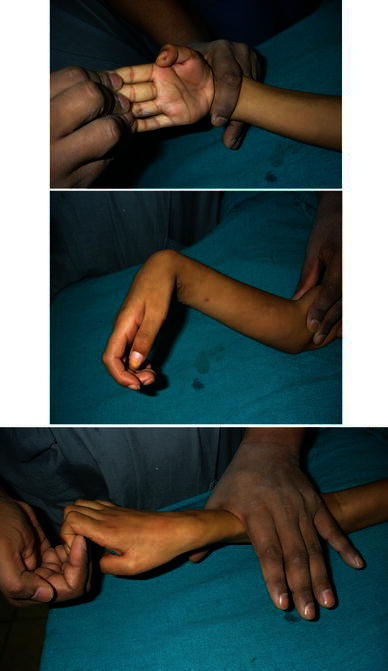
Pre-op clinical photographs (case 1)

**Fig. 2:**
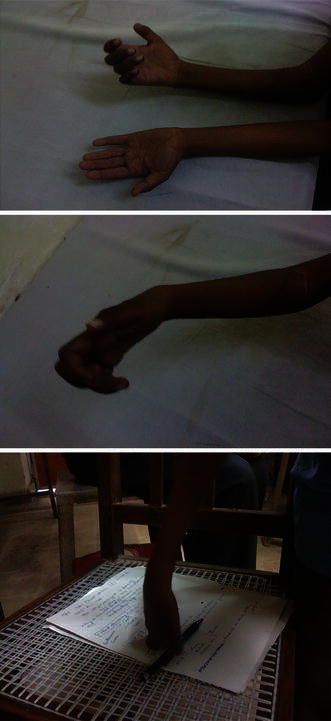
Pre-op clinical photographs (case 2)

Selection criteria for the study included patients with a moderate degree of Volkmann’s ischemic contracture, a positive Volkmann’s sign, no severe contracture of the skin in the forearm, no fixed contractures of the joints of the affected limb, a willingness to take part in the study, and the ability of the patient to understand and perform the postoperative physiotherapy exercises (Figs. 1, 2).

Once the selection criteria had been satisfied, the patient had the procedure, its outcome, complications, and the prolonged rehabilitation protocol that had to be followed for optimal results explained to them. The patients were included in the study after they provided written, informed consent. All patients had preoperative X-rays of the forearm and hand in AP and lateral views. Nerve conduction studies of the ulnar, median, and radial nerves and a Doppler study of the upper limb were performed.

**Fig. 3:**
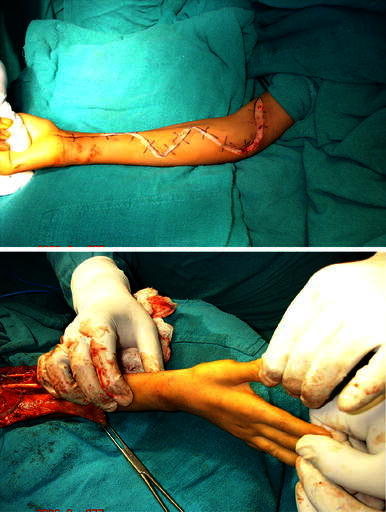
Per-op photographs (case 1)

All of the patients were operated on under general anesthesia. A zigzag-shaped (Fig. 3) skin incision was made from the middle of the lower one-third of the upper arm to the lower one-third of the middle of the forearm. The subcutaneous tissue was separated from the deep fascia on the ulnar and radial sides of the incision. The ulnar nerve was isolated at the level of the elbow and transposed anteriorly. Systematic, complete operative detachment of the origins of the flexor muscles of the forearm was then undertaken. Muscles were dissected subperiosteally using a scalpel. The origins of the pronator teres, flexor carpi radialis, palmaris longus, and the humeral head of the flexor carpi ulnaris were released, and then the flexor digitorum superficialis was detached. The ulnar head of the flexor carpi ulnaris and the broad origin of the flexor digitorum profundus from the anterior aspect of the ulna were detached. Dissection was carried across the interosseous membrane and the origin of the flexor pollicis longus from the anterior aspect of the radius was released. Care was taken to avoid injury to the interosseous artery, vein, and nerves when detaching the flexors from the interosseous membrane. Muscles were allowed to slide distally 2–3 cm. The incision was extended distally when necessary and the palmar wrist capsule and pronator quadratus were released. Postoperatively, the extremity was immobilized for three weeks with the elbow at 90° flexion, the forearm was supinated, and the wrist and digits were extended. After three weeks, a dorsal dynamic extensor splint was prescribed to the patient. The splint was kept on all day for one month, during which time the patient was asked to perform active flexion and splint-assisted extension. After one month, the duration of the splint was reduced to 12 h (during the night), and isolated active joint movement, passive protected extension of the wrist and fingers, and active composite fist movement were advised in-between. The splint was discarded after three months, and resistive isolated joint movement, resistive hook and straight fist, and resistive composite fist were advised thereafter. Job simulation and gradual heavier use was started after six months.

All patients were followed-up in the hospital at three weeks, one month, three months, and then at six-monthly intervals. All patients had a minimum of two years of follow up. During each visit, the operated extremity was examined for hand dexterity, grip power, and sensibility. For hand dexterity, the Sollerman hand function test was used, which includes several tasks based on the most common handgrips [9]. The test was originally developed to evaluate hand function in tetraplegics, but it has recently been increasingly used in other patients [10–13]. For the present study, three of the most relevant tasks for assessing fine manipulation of the hand (picking up coins from a purse, putting nuts on bolts, and doing up buttons) were selected. The range was 0–4 for each task. “0” indicates that the task cannot be performed and “4” indicates that the task is completed without any difficulty within 20 s and with the prescribed handgrip of normal quality. The total score is the sum of all three items (0–12).

**Fig. 4:**
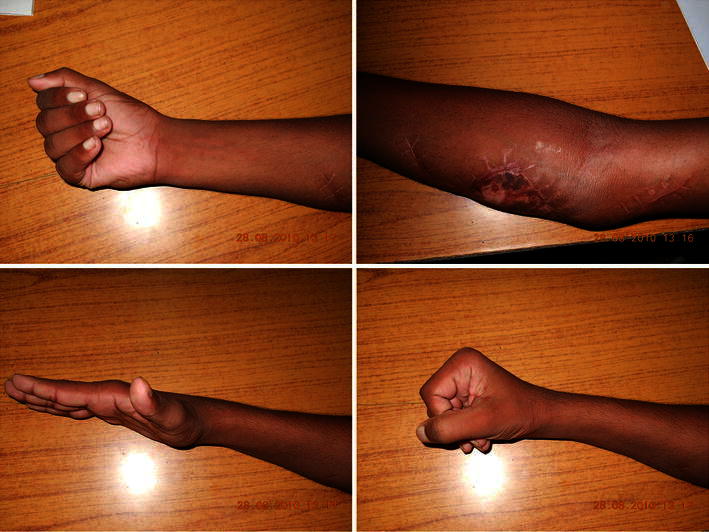
Five-year follow-up clinical photographs (case 1)

**Fig. 5:**
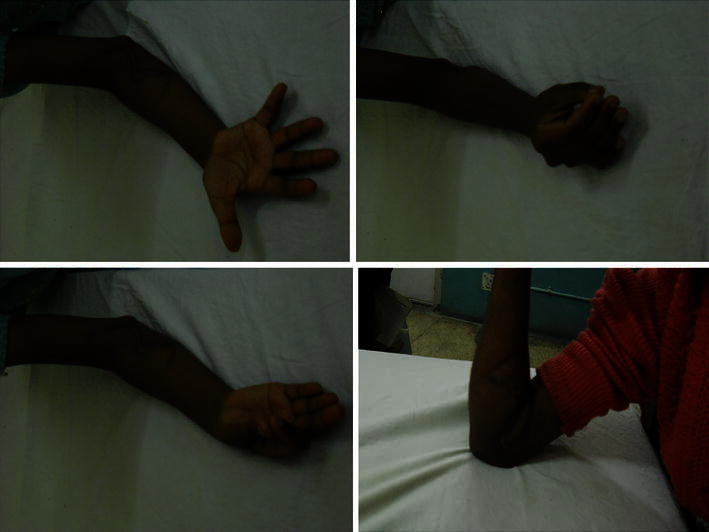
Post-op clinical photographs after 3.5 years (case 2)

Grip power was determined using a Jamar dynamometer [14]. Three trials were performed alternately with each hand, and the mean value was calculated for each hand. The end result was expressed as a percentage of the performance achieved with the uninjured hand.

Sensibility was assessed by means of the Semmes–Weinstein monofilament test with a green colored filament (filament marking 2.83, diameter 0.12 mm). While patients were unable to observe their hands, the monofilament was used at autonomous sites of the radial, ulnar, and median nerves in an alcohol-wiped hand. The SW monofilament was pressed perpendicular to the test site with enough pressure to bend the monofilament for 1 s. Patients were asked to answer yes or no regarding whether they felt the pressing of the monofilament. If a patient did not perceive the filament at all three autonomous zones he was given a score of zero; if he could only perceive the radial autonomous zone he was given a score of one, and a score of two was assigned upon sensing all three autonomous zones.

At the end of the study, the results were graded as either good, fair, or poor by taking into account the dexterity score, the hand grip strength, the sensibility, and the appearance of the hand (Table 2) (Figs. 4, 5).

**Table 2:** Criteria for grading the results at the end of the study

	Dexterity score	Hand grip strength (affected hand/normal hand) × 100	Sensibility score	Appearance of the hand (ability to extend the fingers with the position of the wrist)
Good	≥9	≥75	2	In slight dorsiflexion
Fair	5–8	51–74	1	In neutral position
Poor	≤4	≤50	0	In palmar flexion

Improvements in the dexterity score, hand grip, and sensibility from the preoperative levels were assessed. The postoperative values were compared with the preoperative values using a paired *t* test (Table 3).

**Table 3:** Improvements in grip strength, range of motion, and sensibility

S. no.	Hand grip (%)		Dexterity score		Sensibility score	
	Pre-op	Post-op	Pre-op	Post-op	Pre-op	Post-op
1	17	84	5	11	2	2
2	15	80	6	12	2	2
3	5	80	5	12	2	2
4	0	63	3	9	1	2
5	0	83	3	8	2	2
6	0	20	1	4	1	1
7	0	70	3	9	2	2
8	0	65	2	7	2	2
9	18	79	5	11	2	2
10	6	75	7	12	2	2
11	8	85	6	11	2	2
12	10	78	4	8	2	2
13	0	65	5	10	1	2
14	8	78	7	12	2	2
15	12	85	5	12	2	2
16	0	85	6	10	2	2
17	0	84	5	11	1	2
18	16	80	5	10	2	2
19	20	87.5	5	12	1	2
Mean ± SD	7.10 ± 7.33	75.07 ± 15.24	4.63 ± 1.60	10.05 ± 2.14	1.73 ± 0.45	1.94 ± 0.22
Paired *t* values		21.60		22.07		2.19
d.f.		18		18		18
*p* values		<0.001		<0.001		<0.05

## Results

A total of 19 cases (12 males and 7 females) were enrolled on the study, among whom 8 patients who were operated before 2004 were traced from the medical records and analyzed retrospectively, whereas 11 cases were assessed prospectively. The average age at the time of presentation was 18 years (range 3–25 years). Tight external splintage for injuries around the elbow and forearm was the primary factor, present in 17 cases, while 2 cases had a history of crush injury. Supracondylar fracture was the underlying injury in 12 cases, whereas fractures of the forearm bones were present in 5 cases. Three patients were operated on within 9 months, whereas others were operated on between 1 and 17 years after the injury.

Fifteen patients had hand dexterity scores of >8 (in the good range) when assessed by the Sollerman hand function test, whereas one patient had a score of 4. The postoperative dexterity score varied from 4 to 12, with an average of 10.05, compared to the average of 4.63 preoperatively.

Most of the patients (14 cases) were able to achive grip strengths in the good range (i.e., within ≥75 % of the normal limb). The hand grip strength varied from 20 to 87.5 %, with an average of 75.07 %. Preoperatively, most of the patients were unable to perform the test due to the deformity and poor grip. Eighteen out of 19 had a sensibility of 2, whereas one patient had a sensibility score of 1 when tested by the Semmes–Weinstein monofilament. Fifteen patients had good appearance of the hand, with the ability to extend the fingers in slight dorsiflexion of the wrist, while one patient had a poor appearance of the hand. At the end of the study, 14 patients had good hand function, four had fair, and one had poor results.

None of the patients had a serious complication. Wound dehisence was observed in two patients, which healed by secondary intention. The overall functional results were not affected by this, and the patients showed good outcomes in all of the variables. One patient with poor dexterity and grip strength required a secondary tendon transfer procedure. Two patients developed paresthesias in the distribution of the ulnar nerve, which improved on symptomatic treatment. One patient had diminished sensibility along the ulnar nerve distribution along with Volkmann’s ischemic contracture, which persisted even after the operation.

The functional improvement was assessed by comparing the postoperative values of the three factors (i.e., dexterity score, hand grip status, and sensibility score) with the corresponding preoperative values using a paired *t* test. The mean preoperative value for the hand grip score was 7.15 ± 4.33, which increased to 75.07 ± 15.24 postoperatively, indicating a significant improvement in hand grip status (*p* < 0.001). Similarly, a significant improvement was also observed in the dexterity score, where the average postoperative value improved from 4.63 ± 1.60 to 10.05 ± 2.14 (*p* < 0.001). The sensibility score at the preoperative stage for these patients was 1.73 ± 0.20, which significantly improved after the operation (*p* < 0.05).

## Discussion

Despite our improved understanding of the pathogenesis, diagnostics, and treatment methods of compartment syndrome, the extremely disabling condition known as Volkmann’s ischemic contracture still occurs. The treatment of an established contracture is complicated and depends on the severity of the infarction and the affected muscle and nerve tissue. For the patient and the attending surgeon, knowledge of the long-term outcome of treatment for patients with Volkmann’s ischemic contracture is indispensable for planning and treatment.

Tight external splintage for supracondylar fractures of the humerus was the most common cause of the ischemic contracture in our study. Sundararaj et al. [15], in his study of 196 cases of Volkmann’s ischemic contracture, also reported that tight external splintage was the main causative factor. When considering individual injuries, supracondylar fractures were reported to be the major cause of ischemic contractures in studies by Reigstad et al. [16], Tsuge [8], Eichler [17], and Ultee [18]. Fractures of the forearm bones were found to be the most common cause in a study performed by Chuang et al. [19], in which strategies for preventing Volkmann’s ischemic contracture were evaluated. Other rare causes mentioned in the literature [16, 17, 19] include intoxication, infusion of hypertonic dextrose extravenously, chemotherapy perfusion for therapy of malignant cancers, and following excision of congenital radioulnar synostosis.

Page [20] stated in 1923 that if the muscles are active but extremely contracted, one should perform the disinsertion of the forearm muscles. This method was popularized and improved by Scaglietti [21], and introduced in France by Gosset [22]. It has been a widely used procedure for Volkmann’s ischemic contracture [8, 23–27], and has been shown to be more effective than infarct excision alone for obtaining a lasting correction [7, 8, 20]. Tsuge [8], while evaluating the treatment of established Volkmann’s ischemic contractures, performed muscle slide operations in 14 patients and observed good results in all of them, without any recurrence. Eichler et al. [17], while reviewing the treatment of ischemic contractures at Mayo Clinic, reported 13 patients who underwent muscle slide operations. Nine of the 13 patients showed excellent correction. In another study by Dianming et al. [28], seven cases of Volkmann’s ischemic contracture underwent muscle slide operations. In their study, the results for four of those cases were excellent (S 3M 4), the results for two were good (S 3M 3), and those for one were fair (S 2M 2). We evaluated the functional results using three factors—dexterity, grip strength, and sensibility. Significant improvements in all three factors were observed during the postoperative period when analyzed using the paired *t* test. The better results seen in our study can be attributed to our selection of cases of only moderate severity and a more extensive and rigorous postoperative physiotherapy protocol.

The merits of the muscle sliding operation include its simplicity [8, 17, 20], its ability to correct the deformity in one operation, and the potential to perform tendon transfers as a secondary procedure. However, this method has also been criticized for its unpredictability of correction, risk of recurrence of deformity with growth, as well as the possibility of overcorrection and the resulting decrease in grip strength [23–27]. Most of these problems arise due a lack of proper patient selection, either inadequate or too traumatic release of muscles, and a less focused postoperative physiotherapy protocol. If the surgery is performed adequately and postoperative procedures such as dynamic splints are used correctly, the risk of recurrence of the contracture is not high, and good recoveries of sensation and intrinsic muscle function can be achieved [20, 24, 29].

The main determinant of good functional results in Volkmann’s ischemic contracture is the recovery of hand function [30]. Other important factors include a good range of passive joint movement and adequate hand sensibility. Most of the studies undertaken to date have evaluated their results on the basis of range of motion, sensibility, and motor power [8, 17, 18, 28]. We determined the functional results based on hand dexterity along with the other accepted variables. Adding hand function as an extra criterion to our study improved its ability to detect functionally good results.

Because of the fact that Volkmann’s ischemic contracture is a relatively rare condition, our study population was too small to be able to provide a statistically relevant correlation between our method of treatment and functional outcome. However, our results do indicate some correlation. Our study results can help with decision making relating to the treatment of patients with Volkmann’s ischemic contracture, and can serve as a template for further research on this subject.
